# Mycobiota profile, phenology, and potential toxicogenic and pathogenic species associated with stored groundnuts (*Arachis hypogaea* L.) from the Volta Region, Ghana

**DOI:** 10.1002/fsn3.2719

**Published:** 2022-01-10

**Authors:** Nii Korley Kortei, Rachel Adinorkie Tetteh, Michael Wiafe‐Kwagyan, Denick Nii Kotey Amon, George Tawia Odamtten

**Affiliations:** ^1^ Department of Nutrition and Dietetics School of Allied Health Sciences University of Health and Allied Sciences Ho Ghana; ^2^ Department of Plant and Environmental Biology College of Basic and Applied Sciences University of Ghana Legon Ghana

**Keywords:** *Fusarium* and *Curvularia* species, Groundnut, mycobiota phenology, mycotoxicogenic and pathogenic *Aspergillus*, *Penicillium*

## Abstract

This study updates the mycobiota resident in groundnut seeds, their phenology during storage with the view to ascertain their occurrence, potential toxigenic species, and pathologically important species in the stored samples. The moisture content of the seeds ranged from 5.7% to 6.5% within the stipulated safe moisture content of 8% for extension of shelf life. Culturing the seeds on mycological media (Sabouraud's Dextrose Agar SDA; Oxytetracycline Glucose Yeast Extract OGYE, Potato Dextrose Agar, PDA) caused a de novo growth of the quiescent spores at 28–30°C for 7–14 days. Fungal population counts on the three media ranged from 2.01 to 2.16 log_10_ CFU/g samples to a final 6‐month count of 1.67–2.60 log_10_ CFU/g. Eighteen different fungal species belonging to ten genera were encountered on the media, namely *Aspergillus, Cladosporium, Curvularia, Fusarium, Penicillium, Trichoderma, Rhizopus, Rhodotorula, Sporendonema*, and *Paecilomyces*. *Aspergillus spp*. (*A. niger, A. flavus, A. fumigatus*, and *A. terreus)* were the most frequently isolated, followed by *Fusarium species (F. oxysporum, F. solani*, and *F. verticillioides), Trichoderma (T. harzianum* and *T. viride), Rhizopus spp (R. oligosporus* and *R. stolonifer)*, and *Penicillium verrucosum*. The species which were seed borne *(A. niger, A. flavus, A. terreus, A. fumigatus, F. solani, F. verticillioides, T. viride, C. herbarum*, and *Curvularia lunata)* were isolated on both surface sterilized and non‐surface sterilized seeds. The phenology of the encountered fungal species generally followed five patterns. The most frequently isolated *Aspergillus niger, A. flavus*, and *A. fumigatus* predominated throughout the 6 months sampling period, while *A. ustus* and *A. terreus* appeared sporadically and disappeared. The early colonizers (*R. oligosporus, R. stolonifer*, and *Paecilomyces*) could not be isolated after 2–3 months owing presumably to stronger antibiosis competition from the *Aspergillus* species. The most predominant *Aspergillus* species initially constituted 36%–48% of the total population but declined to 10%–36% in 6 months. Mycobiota encountered with mycotoxigenic potential and human health importance were *A. niger, A. flavus, A. fumigatus, F. verticillioides*, and *Penicillium verrucosum*. Other species of pathological importance to plants were *Curvularia lunata* and *Fusarium oxysporum*. The practical implications of these findings are discussed.

## Graphical abstract

Mycobiota phenology of toxicogenic fungi that contaminate groundnuts that are an excellent source of nutrients and used extensively to curb malnutrition.

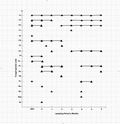

## 1 INTRODUCTION

Groundnut *(Arachis hypogaea)* is an annual leguminous crop, which provides food for humans and serves as feed ingredients for animals. It belongs to the family *Fabaceae* (Leguminosae) and it is known by many local names such as earthnuts, ground nuts, goober peas, monkey nuts, pygmy nuts, and pig nuts (wikipedia.org). Although its origin is in South America, it is cultivated in many countries including: China, India, Mali, Gambia, Senegal, Chad, Ghana, and Nigeria (Yussif, 2016). Groundnut is the 4th most important oilseed crop and the 13th most important crop worldwide (Reddy et al., 2011). In the sub‐Saharan region of Africa, groundnut is considered a valuable crop as it comprises 40% of the world's production area, but contributes about 26% to the groundnut production worldwide (Angelucci and Bazzucchi, 2019). In Ghana, it is grown in almost all the 16 regions but predominates in the Northern, Upper East, Upper West, Oti, and Volta Regions where it is cultivated as a cash crop (Oteng‐Frimpong et al., 2015). It is estimated that the total production nationwide was about 193,200 metric tons from these regions according to Chakuri (2018). In 2015, farmers in Ghana produced 417,000 metric tons from the pulse from 336,000 hectares of land (MoFA‐SRID, 2016). Groundnut is grown throughout tropical Africa as a cash crop particularly in Senegal, Gambia, Nigeria, Sudan, and Ghana (Senghor et al., 2020).

Apart from deriving income from cultivating groundnut, it provides an inexpensive source of high‐quality dietary protein, oil, and mineral elements (Asibuo et al., 2008). The chemical composition of Ghanaian varieties of groundnut is different from what is obtained elsewhere and the vast food preparations incorporating groundnut to improve the protein level has helped in no small measure, in reducing malnutrition in developing countries (Asibuo, Akromah, Adu‐Dapaah, et al., 2008; Asibuo, Akromah, Safo‐Kantanka, et al., 2008).

There are about 21 cultivars of groundnut in Ghana divided into subspecies *hypoaea* and *fastigita*. This division is based on their branch patterns, presence or absence of flowers on the main stem, flower arrangement on leaf axils, etc. (Asibuo, Akromah, Safo‐Kantanka, et al., 2008). Cultivars with flowers on the main stem, sequential branching, and flowering were grouped into the subspecies *fastigita* Waldon and those without flowers on the main stem, alternative branching patterns, and alternate flowering were grouped into subspecies *hypogaea* category (Stalker et al., 2016). Those varieties belonging to the *Arachis hypogaea* Dagomba, F‐mix, Nkatepa, Manipinta, Sinkazie, Kumawu red/early, and Nkate kokoo; and those belonging to *Arachis fastigita* are Baasare, Broni nkatee, Afu, Nkoranza local, Atebubu local, Aprewa, Kintampo local, Shitaochi, Broni, Kamaloo, Kofi Nsarko, Koweka, Broni fufuo, and Florispan runner (Asibuo, Akromah, Safo‐Kantanka, et al., 2008). Oil content of the listed varieties ranged from 33.6% to 54.95% (Asibuo, Akromah, Safo‐Kantanka, et al., 2008). Crude protein ranges from 18.92% to 30.53%; carbohydrates ranges between 6.0% and 24.9%; and potassium, K values range between 1180 and 1693 mg/100 g, Na (19.0–40.0 mg/100 g), Ca (44–134 mg/100 g), and Mg (308–456 mg/100 g). The range of K, Na, and Mg on the 20 cultivars in Ghana was generally higher than the results from other workers (Galvao et al., 1976; Khalil & Chughtai, 1983; Oerise et al., 1974; Oke, 1967). Zinc, Zn, content ranged from 0 to 6.5 mg/100 g with a mean of 5.2 mg/100 g; Cu differed from 0 to 27 mg/100 g with a mean of 1.9 mg/100 g; Fe (0.2–3.7 mg/100 g; mean of 2.8 mg/100 g); and Mn (1.7–2.9 mg/100 g with a mean of 2.1/100 g). All the varieties had appreciable amounts of Zn, Cu, Fe, and Mn except Kintampo local which had no zinc and copper. Same range of values were obtained in findings of Galvao et al. (1976), Khalil and Chughtai (1983), Oerise et al. (1974), and Oke (1967). The amounts of micronutrients in the cultivars reported by Asibuo, Akromah, Safo‐Kantanka, et al. (2008) were nutritionally significant because small quantities are needed by the body (Toomer, 2018).

Matured groundnut seed contains per 100 g edible portion, 6.5 g water, energy 237 KJ (567 kcal); protein 25.8 g; fat 49.2 g, dietary fiber 8.5 g, thiamine 0.64 mg, riboflavin 0.14 mg, niacin 12.1 mg, vitamin B6 0.35 mg, folic acid 240 µg, and vitamin C 0 mg (Burkill, 1995). Essential amino acids in groundnut per 100 g of edible portion are tryptophan (250 mg), lysine (926), methionine (317 mg), phenylalanine (13,337 mg), threonine (883 mg), valine (1082 mg), leucine (1672 mg), and isoleucine (907 mg) (PROTA, 2015; Burkill, 1995). Fatty acids per 100 g edible portion are oleic (27.7 g), linoleic (15.6 g), and palmitic acids (5.2 g) (Ntare et al., 2008). Clearly, groundnut is a rich source of nutrition and does play a significant role in food and nutrition security as a valuable source of protein, fat, energy, minerals, and income‐generating commodity for many poor farmers in Sub‐Saharan Africa and Asia (Diop et al., 2004). However, its rich nutrient source is also the reason for utilization by microorganisms, especially fungi, for further growth and survival both in the field and in storage, thus reducing the market and nutritional values.

There are other human benefits to the consumption of groundnut. For example, consumption of groundnut reduces the risk of cardiovascular diseases, increases serum magnesium, and provides protection against certain human cancers (Alasalvar et al., 2020; Arya et al., 2016; Ros, 2010). Studies by Lokko et al. (2007) have demonstrated that groundnut lowers the total cholesterol and triacylglycerol concentration (Brostow et al., 2011). Lilly et al. (2018) showed that groundnut can be used to control type 2 diabetes (groundnut butter has low glycemic index and glycemic load). It contains high levels of vitamin E which helps in protecting one against Alzheimer's disease and age‐related cognitive decline (Morris, 2004). Recently, Mupunga et al. (2017) showed that groundnut butter is used as a main ingredient in the ready‐to‐use therapeutic food (RUTF),—an energy and protein—dense paste which meets the nutritional needs of population at risk of malnutrition.

Groundnut production is faced with a plethora of constraints, namely drought, fungal (rusts and leaf spots), bacterial, viral diseases, as well as insect pests (leaf miners and aphids) (Okello et al., 2010). Prominent among these diseases is the infection of groundnut seeds by species of *Aspergillus* and *Penicillium* producing mycotoxins. Mycotoxigenic fungi can invade and produce potent mycotoxins in seeds of groundnut prior to harvest, during harvest, and in storage after harvest. Recently, Bediako, Ofori, et al. (2019) reviewed the predisposing factors and management of aflatoxin contamination of groundnut. The genus *Aspergillus* is subdivided into seven (7) subgenera, which are further divided into sections (Klich, 2002). *Aspergillus* section *flavi* commonly referred to as *Aspergillus flavus* group (Amaike & Keller, 2011) belonging to the subgenus *Circumdati* has gained popularity due to its ability to produce toxins. *Aspergillus oryzae, A. sojae*, and *A. tamarii* form the non‐aflatoxigenic group while *A. flavus, A. parasiticus*, and *A. nomius* constitute the aflatoxigenic group of the section *Flavi* (Rodrigues et al., 2007). *A. nomius* is considered a minor pathogen and not of practical importance (Pitt, 2000). *A. flavus* and *A. parasiticus* are, on the other hand, economically and agronomically important species which infect and produce aflatoxin (B1, B2, G1, and G2) in agricultural crops prior to harvest, or during storage (Yu et al., 2004), under suitable environment during pre‐ or post‐harvest operations.

Aflatoxigenic fungi invade and produce aflatoxin in pods and seeds of groundnut prior to harvest, during harvest, and after harvest. The inoculation and colonization of *A. flavus/A. parasiticus* with initial inoculation from the soil bank of resident spores depend on the fungal population, temperature, relative humidity, insect infestation, and moisture content of the soil (Bediako, Ofori, et al., 2019; Gnonlonfin et al., 2013; Guchi, 2015). Complex interactions among the listed factors occur which could result in significant invasion of groundnut seeds (Gnonlonfin et al., 2013; Pandey et al., 2019). Furthermore, high soil temperature and late‐season drought stress are two important factors which occur concurrently to promote pre‐harvest infestation and aflatoxin contamination of groundnut (Bediako, Ofori, et al., 2019). Post‐harvest aflatoxin contamination of groundnut is, however, influenced by high humidity and high temperature during storage (Guchi, 2015). Aflatoxin contamination results in the damaging of grains and oilseed and depletion of their nutritional value (Jolly et al., 2009). Prolonged consumption of aflatoxins has been associated with impaired immune function (immunosuppressive effect), malnutrition and stunted growth in children, disabilities, and death (Achaglinkame et al., 2017; Bbosa et al., 2013). Other adverse health effects of intake of aflatoxins include liver cirrhosis, hepatitis B and C infection, and liver cancer (Bbosa et al., 2013).

Fumonisin, another type of fungal toxin, is produced by several species of the genus *Fusarium* such as *Fusarium verticillioides* and *Fusarium moniliforme*, which can infect many crops, especially cereals such as maize, wheat, and barley (Zeng et al., 2020). Cinar and Onbaşı (2019) also stated that 28 types of fumonisin have been identified in foods including groundnuts and have been divided into four groups: Fumonisin A (A1, A2, and A3), Fumonisin B (B1, B2, and B3), Fumonisin C (C4, C3, and C1), and Fumonisin P (P1, P2, and P3). Frequently ingested food or feed containing fumonisin cause diseases such as equine leukoencephalomalacia, porcine pulmonary edema, and possibly kidney and liver cancer (Riley and Merill, 2019). The co‐occurrence of fumonisin and aflatoxins in foods or feed is common.

Generally, there is a safe moisture content for all grains, pulses, and legumes which can prolong the shelf life of the seeds. Moisture content of 10% or higher after harvest predisposes groundnut to aflatoxin contamination (Bediako, Ofori, et al., 2019; Waliyar et al., 2015). Therefore, timely drying and maintenance of safe moisture level would achieve effective content of post‐harvest aflatoxin contamination (Torres et al., 2014). A positive correlation between kernel moisture content of groundnut and aflatoxin production was found by Kaaya and Kyamuhangire, (2006), cowpea, (Houssou et al., 2009) and maize (Hell et al., 2000). The water activity (a_w_) is a measure of the amount of free humidity in a product and the water vapor pressure of a substance divided by the vapor pressure of pure water at the same temperature. Water activity beyond 0.85a_w_ at 25°C provides a conducive environment for fungal growth and spore germination (Hassane et al., 2017; Lasram et al., 2010). The optimum water activity (a_w_) for growth of *A. flavus* is 0.996 a_w_ and the minimum is 0.80–0.82 a_w_ (Giorni et al., 2012; Northolt et al., 1977). To prevent aflatoxin formation in groundnut, the seeds must be dried to or below a_w_ 0.83 after harvest.

Proper storage of groundnut is one maintained under clean, dry conditions with low kernel moisture content about 8% and low temperature with protection from insect infestation to avoid contamination and aflatoxin formation (Torres et al., 2014). In Ghana, Awuah and Ellis (2002) observed that groundnuts dried to 6.6% were devoid of fungal infection for 6 months irrespective of the storage method, while at 12% and stored in only jute bags with 3% (w/w) Syzygium aromaticum powder as a protectant, effectively inhibited *A. parasiticus* infection. They recommended that for post‐harvest management of aflatoxin contamination, maximum moisture contents 9% mc for unshelled groundnuts and 7% for shelled groundnuts in Ghana should be maintained during storage at 70% ERH and 25–27°C. The calculated moisture content to maintain 90% germination for shelled groundnut seeds storage for 6 months at 15°C was 8.09%mc for variety Hanoch and 7.9% for Congo variety (Navarro et al., 1989). There is no information on the Ghanaian groundnut seed varieties in this respect.

Bediako, Dzidzienyo, et al. (2019) recently reported the incidence of fungi on unspecified varieties of groundnut in four growing regions (Ashanti, Upper East, Upper West, and Northern) of Ghana. *Aspergillus niger* (39.9%) and *A. flavus* (26.3%) were predominant species recovered, respectively, from 73.3% to 83.3% of 60 groundnut samples. *A. flavus* was found in equal proportion in the four regions studied. Other fungal species identified were *Collectotrichum* (13.3%), *Rhizopus* (14.8%), *Penicillium* (5.4%), *Curvularia* (0.2%), and *A. ochraceus* (=*A. alutaceus*). Awuah and Kpodo (1996) found *A. flavus* (12.8%–31.7%), *A. parasiticus* (0.24%), *A. niger* (34.0%), *A. candidus* (1.45%), *A. tamarii* (3.93%), *A. alutaceus* (=*A. ochraceus*; 5.26%), *Fusarium spp* (1.7%), *Penicillium spp*. (5.19%), *Mucor sp*. ( 2.3%), *Trichoderma sp*. (0.2%), *Rhizopus stolonifer* (12%), and unidentifiable fungi (11.72%) on groundnut from 21 selected markets in the then 10 regions of Ghana. According to Waliyar et al. (2007), *Fusarium*, *Chaetomium*, *Alternaria*, *Basidiomycota*, and *Sordaria* affect yield and quality of groundnut worldwide. The environmental conditions where the different groundnut cultivators grow may also influence the structure of the fungal community (Li et al., 2018).

In one of the first detailed study of mycobiota associated with groundnuts in Ghana, Markwei (1976) used two varieties of groundnuts (Kumawu Red and Florispan Runner) cultivated in the Volta Region in Ghana. The following results were obtained:

### 1.1 *Florispan runner* (Dabala)

*Aspergillus spp., Alternaria sp., Botryodiplodia theobromae, Cladosporium herbarum, Chaetomium sp., Curvularia sp., Fusarium sp., Helminthosporium sp., Macrophomina, Mucor sp., Neurospora crassa, Nigrospora, Papulospora,* 12 *Penicillium* species*, Piptocephalis sp., Rhizopus oryzae, Trichothecium roseum, Trichoderma viride*, and sterile mycelium (Mycelia sterilia)*, Syncephalastrum racemosum*, and *Verticillium*.

### 1.2 *Florispan runner* (Sogakope)

*Aspergillus species, Arthrobotrys anoidea, Chaetomium, Cladosporium, Curvularia, Fusarium, Gliocladium roseum, Helminthosporium sp., Macrophomina, Mucor, Neurospora crassa, Nigrospora, Papulospora,* 12*Penicillium spp., Piptocephalis, Rhizopus oryzae, Sclerotium rolfsii, Verticillium*, and Mycelia sterilia.

### 1.3 *Kumawu red* (Dabala)

*Aspergillus sp., Botryodiplodia theobromae, Chaetomium, Cladosporium, Collectotrichum, Curvularia, Fusarium, Macrophomina, Mucor sp., Penicillium, Periconia, Pestalotia, Phoma, Piptocephalis, Rhizopuz oryzae, Trichoderma viride*, and Mycelia sterilia.

The predominant genera of the florispan runner seeds were *Aspergillus* and *Penicillin* followed by *Chaetomium*, *Fusarium*, *Macrophomina*, *Mucor*, *Papulospora*, and *Rhizopus*. *Aspergillus* and *Penicllium* were again the outstanding genera of fungi isolated from the Kumawu red seeds followed by *Chaetomium*, *Macrophomina*, and *Mucor* (Markwei, 1976). In most instances, three pattern of phenology were shown. *A. flavus* and *A. niger* increased with storage time; on the other hand, *Macrophomina* and *Penicillium* declined with prolonged storage (Markwei, 1976). Finally, *Chaetomium* population increased for 3–4 months and then declined. The same was true for *Mucor sp*. Many species did not show any trend and there was marked fluctuations. Presumably, the unique environmental conditions in the farms where the different cultivars (Florispan runner and Kumawu Red) were cultivated influenced the structure of the fungal community (Li et al., 2018).

The objective of this present study was to update the mycobiota of the Kumawu red varieties cultivated in the Volta Region of Ghana with the view to updating the mycobiota profile and their phenology and to identify potential mycotoxigenic and pathogenic species associated with stored groundnuts cultivated in the Kpetoe District of the Volta Region of Ghana.

## 2 MATERIALS AND METHODS

### 2.1 Sample collection

Twenty groundnut samples (variety: Kumawu red; mass 0.5 kg; deshelled) were purchased from a farm at Kpetoe (6° 33 N 0° 43E) in the Volta Region of Ghana, 23 km Southeast of Ho. The samples were transported in standard jute bags to the laboratories of the University of Health and Allied Sciences, Dave, Ho, in the Volta Region and the Department of Plant and Environmental Biology, University of Ghana, Legon, where the experiments were carried out. The sample of groundnut was stored in the laboratory under normal environmental regime of 75%–80% ERH and 28–32°C. The seeds (500 g) were sampled monthly from the top (0–20 cm), middle (20–40 cm), and bottom (40–60 cm) of the jute bags and then pooled together.

### 2.2 Mycobiota determination

#### 2.2.1 Blotter method

A modified method of de Tempe (1967) and Limonard (1967) was employed. Ten seeds, surface sterilized with 1% sodium hypochlorite for 15–20 min and 10 non‐surface sterilized seeds were placed on Whatman's filter paper in 9 cm sterile Petri dishes and then moistened with about 10 ml sterile distilled water. There were 25 replicates (250 seeds) for each treatment. The plates were incubated for up to 14 days at 28–30°C. The following quantitative assessments were made:

- Percentage germination of seed initially and after 6 months in the jute bag
- Percentage of seeds infected with fungi
- Percentage of seeds infected with a particular fungus species
- The total number of fungal colonies on the grains

#### 2.2.2 Solid medium method

Surface sterilized seeds (with 1% sodium hypochlorite) and non‐surface sterilized seeds were placed directly on each Petri plate containing Solid Potato Dextrose Agar (PDA, Oxoid), Oxytetracycline Glucose Yeast Extract Agar (OGYE, Oxoid), and Sabouraud's Agar (SA, Oxoid). Each medium was amended with chloramphenicol (500 mg/L). There were 20 replicates for each treatment and medium used. Sodium hypochlorite treatment was used with the view to reducing or removing completely, external saprophytes, which compete with resident pathogens within the seed. Each treatment and plates were incubated at 28–32°C for a period of 7 days until fungi grew.

#### 2.2.3 Estimation of viable fungal colonies

Exactly 50 g of sample was weighed into 100‐ml 0.1% peptone water in 250‐ml Erlenmeyer flasks using electronic weighing balance (OHAUS^®^) with a readability of 0.01 g. The samples were shaken in a Gallenkamp Orbital Shaker (140 rev/min) for 30 min. From the stock suspension, decimal serial dilutions up to 1:10^3^ were prepared. Exactly 1 ml aliquots of each dilution level were dispensed into 20 ml of media Potato Dextrose Agar (PDA), Sabouraud's Dextrose Agar (SDA), and Oxytetracycline Glucose Yeast Extract Agar (OGYE) as previously outlined by Kortei et al. (2018) and Odamtten et al. (2018). There were triplicate samples for each media and dilution level. The plates were incubated at 28–30°C for up to 7 days.

### 2.3 Fungal enumeration and identification

Fungal enumeration was done using a colony counter (STAR 8500 Funke Gerber) and then calculated as colony‐forming unit per gram sample (CFU/g). Data obtained in the standard units were transformed into the logarithmic form and presented as log_10_ CFU/g sample (Kortei et al., 2018; Odamtten et al., 2018).

Molds and yeast that appeared were identified by their cultural and morphological characteristics using standard identification manuals (Samson and Reenen‐Hoekstra, 1988; Samson et al., 1995).

### 2.4 Determination of moisture content (MC)

The moisture content (MC) of the groundnut seeds used at each sampling interval was determined using a modification of the oven dry weight method of Basoglu and Uylaser (2000). The seeds were milled using Waring Blender. Ten grams of flour was dried for 24 h in an oven (Gallen kamp) at 105°C and cooled in a desiccator. The sample was weighed using an Accu Lab ALC‐150.3 balance and MC determined using the following equation:

$$\text{MC}=\frac{W2‐W3}{\text{W2}‐\text{W1}}\times 100$$

where W1 is weight of empty Petri dish

W2 is weight of sample + Petri dish before drying

W3 is weight of sample + Petri dish after drying

### 2.5 Data analysis

Data recorded and graphical representations were made using Microsoft Excel for Windows 9. Means were exported to Graphic Software System, STCC, Inc., USA, for analysis. Comparisons of means were performed using Duncan's multiple‐range test. A *p*‐value of <.05 was considered as significant at all points of analysis.

## 3 RESULTS

### 3.1 Mycobiota determination

**FIGURE 1:**
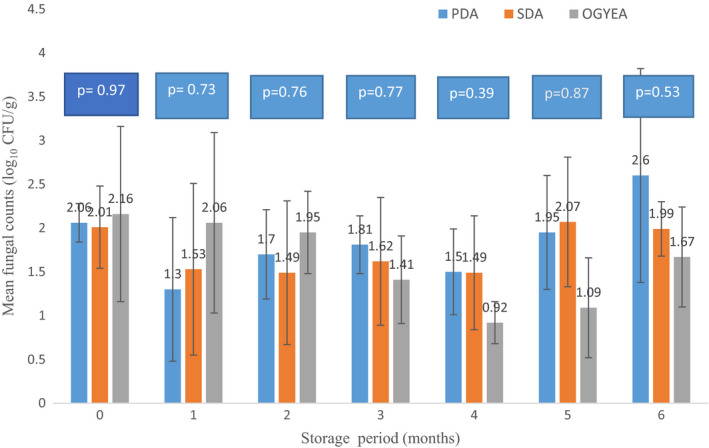
Comparative population of resident fungi (log_10_ CFU/g sample) in *Arachis hypogaea* L stored for up to 6 months and incubated on PDA, SDA, or OGYE after each sampling interval at 26–32°C. Abbreviations: PDA, Potato Dextrose Agar; SDA, Sabouraud Dextrose Agar; OGYE, Oxytetracycline Glucose Yeast Extract Agar

**FIGURE 2:**
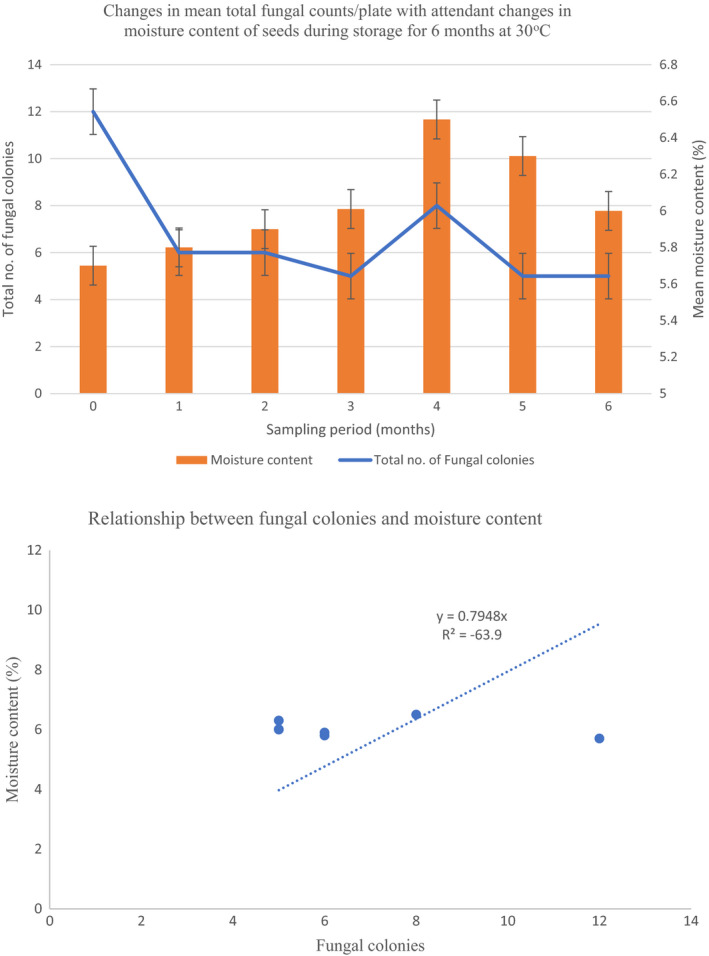
(a) Changes in mean total fungal counts/plate with attendant changes in moisture content of seeds during storage for 6 months at 30°C. (b) Relationship between fungal colonies and moisture content

Figure 1 summarizes results obtained using three media PDA, SDA, and OGYE for the isolation of fungal isolation of fungal spores. On the three media, the fungi behaved differently and increased marginally from a population of log_10_ 0.9–2.6 log_10_ CFU/g in 6 months. There were no significant (*p* > .05) differences observed in the 6 months storage period (Figure 1). During this same period of sampling, the total number of fungal colonies decreased from 12 to 5 colonies except for a spike to 8 colonies/plate after 4 months (Figure 2a). Correspondingly, the mean moisture content of seeds in the storage bags varied marginally between 5.7 ± 0.21% and 6.5 ± 0.04% (Figure 2a) from top to bottom as shown in Table 1. There was a poor fit *R* ^2^ = −63.9 to the linear equation *y* = 0.7948*x*, as shown in Figure 2b.

**TABLE 1:** Moisture content (%) of groundnuts stored over the entire 6 months storage period

% Moisture content of seed in bag after (months)							
Sampling position in the bag	0	1	2	3	4	5	6
Top (0–20 cm depth)	5.9	6.1	5.8	6.0	6.4	6.3	5.8
Middle (20–40 cm depth)	5.8	5.7	6.2	6.8	6.5	6.5	6.3
Bottom (40–60 cm depth)	5.4	5.5	5.9	6.3	6.5	6.1	5.9
Mean ± Std	5.7 ± 0.21^c^	5.8 ± 0.25^c^	5.9 ± 0.17^bc^	6.4 ± 0.33^a^	6.5 ± 0.04^a^	6.3 ± 0.16^ab^	6.0 ± 0.22^bc^

Note

Means with same superscripts letters in a row are not significantly different (*p* < .05)

Eighteen different fungal species belonging to ten genera were encountered; namely *Aspergillus, Cladosporium, Curvularia, Fusarium, Penicillium, Trichoderma, Rhizopus, Rhodotorula, Sporendonema*, and *Paecilomyces* (Table 2). *Aspergillus* species *(A. niger, A. flavus*, and *A. terreus)* were most frequently encountered followed by *Fusarium (F. oxysporum, F. solani*, and *F. verticillioides), Trichoderma (T. harzianum* and *T. viride), Rhizopus (R. oligosporus* and *R. stolonifer)*, and a single species of *Penicillium (P. verrucossum*) (Table 2). Surface sterilization removed some contaminants. However, species which appeared on the unsterilized seeds were almost same as recorded on the sterilized seeds (Table 2), indicating that they were seed borne from the field. These were *A. niger, A. flavus, A. terreus, A. fumigatus, F. solani, T. viride, C. herbarum, F. verticillioides*, and *F. oxysporum*.

**TABLE 2:** Pooled data of total fungi isolated from shelled groundnut (Kumawu red variety) stored for 6 months under laboratory conditions (ERH 75%–85%; 28–32°C)

*Aspergillus niger* Van Tiegher^SS, 0, 1, 2, 3, 4, 5, 6^
*A. flavus* Link^SS, 0, 1, 3, 4, 5, 6^
*A. fumigatus* Fresen^SS, 0, 2, 3, 4, 5, 6^
*A. ustus* (Banier) Thom and Church^3^
*A. terreus* Thom^SS^
*Cladosporuim herbarum* (Pers.) Link^SS, 0, 1, 2^
*Curvularia lunata* Boedjin^0, 2^
*Fusarium oxysporum* Sehldt.^1, 3, 4, 5, 6^
*F. verticillioides* (Sacc.) Nirenberg^0^
*F. solani* Von Martius^SS, 0^
*Penicillium verrucosum* Dierckx.^0, 1, 2, 4, 5, 6^
*Paecilomyces variotii* Bainier^0, 1, 6^
*Trichoderma harzianum* Rifai^2^
*T. viride* Pers Ex Fr.^SS, 4, 5^
*Rhizopus oligosporus* Saito^0^
*R. stolonifera* (Ehrenb) Vuill.^1, 4^
*Rhodotorula mucilaginosa* (A. Jorg) F.C. Harrison^4^
*Sporendonema casei* Desm^1, 4^

Note

Superscripts show treatments in which fungal species appeared in.

### 3.2 Phenology of the fungal species in the seeds

**FIGURE 3:**
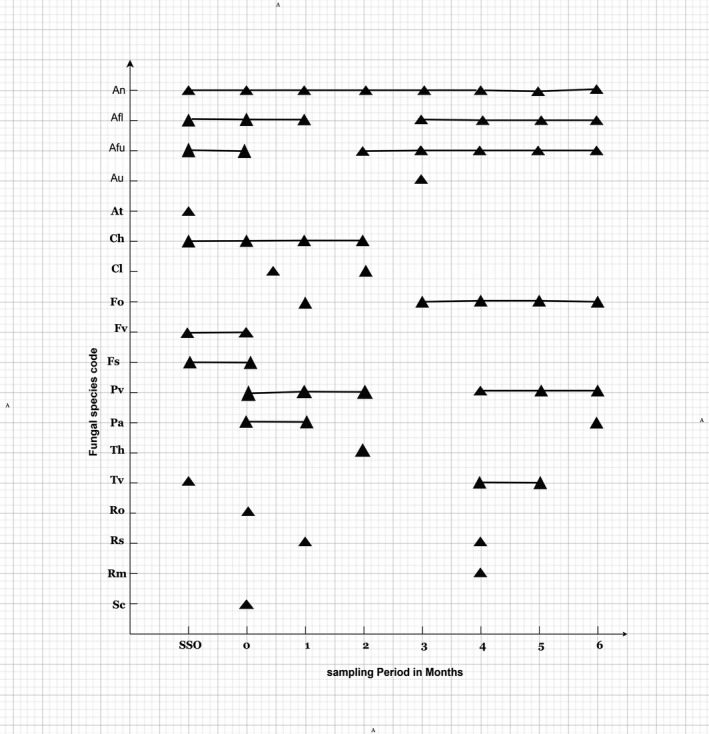
Phenology of resident fungi in groundnut (*Arachis hypogaea*) stored in the laboratory for up to 6 months (SSO—Surface Sterilized; 0—initial, non‐surface sterilized)

The phenology of the fungal species isolated from the seeds followed interesting trends (Figure 3):

- The most frequently isolated *Aspergillus* species predominated throughout the sampling period. *A niger* was isolated at each sampling interval followed by *A. flavus* and *A. fumigatus* in all but one sampling time.
- The occurrence of *A. ustus* and *A. terreus* can be described as sporadic then disappeared thereafter.
- *Cladosporium herbarum* was isolated at the initial stages and persisted for 2 months and thereafter could not be isolated.
- *F. oxysporum* was not initially isolated but subsequently could be detected from 3 to 6 months.
- *Penicillium verrucosum* was isolated throughout the sampling period except the 3rd month akin to *A. flavus* and *A. fumigatus*.
- The occurrence of the rest of the fungal species can be described as early colonizer (e.g., *Rhizopus oligosporus, R. stolonifer, S. casei*, and *Paecilomyces*), which disappeared with stronger competition.

There was, therefore, generally five patterns of infection in the phenology of the contaminating fungal species. The corresponding percentage occurrence of the individual species contaminating the groundnut seeds is summarized in Table 3. The most predominant *Aspergillus* species (*A. niger, A. flavus*, and *A. fumigatus*) initially recorded 36%–48% occurrence declining with time to 10%–15% in 6 months; *A. niger* was the most dominant contributing 10%–48% followed by *A. flavus* (10%–36%) and *A. fumigatus* (10%–36%) (Table 3). Percentage occurrence of *P. verrucosum* ranged from 10% to 20% in 6 months. The remaining species (*Rhizopus*, *Rhodotorula*, and *Sporendonema*) showed sporadic occurrence ranging from 2% to 10%. There were more fungal species isolated from the non‐sterilized seeds (12) as compared to eight on the sterilized seeds and the fungal diversity oscillated between 5 and 8 during the observation period (Table 3).

**TABLE 3:** Percentage occurrence of individual contaminating fungal species in groundnut (Kumawu Red) from Kpetoe, Volta Region, stored for 6 months

Fungal species	Percentage (%) occurrence after (months)							
	SS*	0	1	2	3	4	5	6
*Aspergillus niger*	48.0	41.2	20.0	20.0	15.0	10.0	10.0	10.0
*A. flavus*	36.0	15.9	32.5	‐	10.0	25.0	25.0	20.0
*A. fumigatus*	36.0	38.2	–	25.0	30.0	10.0	10.0	15.0
*A. ustus*	–	–	–	–	15.0	–	–	–
*A. terreus*	4.0	–	–	–	–	–	–	–
*Cladosporium herbarum*	4.0	8.8	10.0	15.0	–	–	–	–
*Curvularia lunata*	–	10.0	–	5.0	–	–	–	–
*Fusarium verticillioides*	20.7	50.0	–	–	–	–	–	–
*Fusarium oxysporum*	–	–	10.0	–	30.0	20.0	25.0	35.0
*Fusarium solani*	2.7	2.9	–	–	–	–	–	–
*Paecilomyces variotii*	–	10.0	10.0	–	–	–	–	5.0
*Penicillium verrucosum*	–	20.0	10.0	20.0	‐	15.0	20.0	15.0
*Trichoderma harzianum*	–	–	–	15.0	–	–	–	–
*Trichoderma viride*	8.0	–	–	–	–	15.0	10.0	‐
*Rhizopus oligosporus*	–	2.9	–	–	–	–	–	–
*Rhizopus stolonifer*	–	7.5	–	–	–	3.0	–	–
*Rhodotorula mucilaginosa*	–	‐	–	–	–	2.0	–	–
*Sporendonema casei*	–	10.0	–	–	–	–	–	–
Total	8	12	6	6	5	8	6	6

Note

SS*, Initial, Surface Sterilized; O, Initial non‐surface sterilized.

**PLATE 1:**
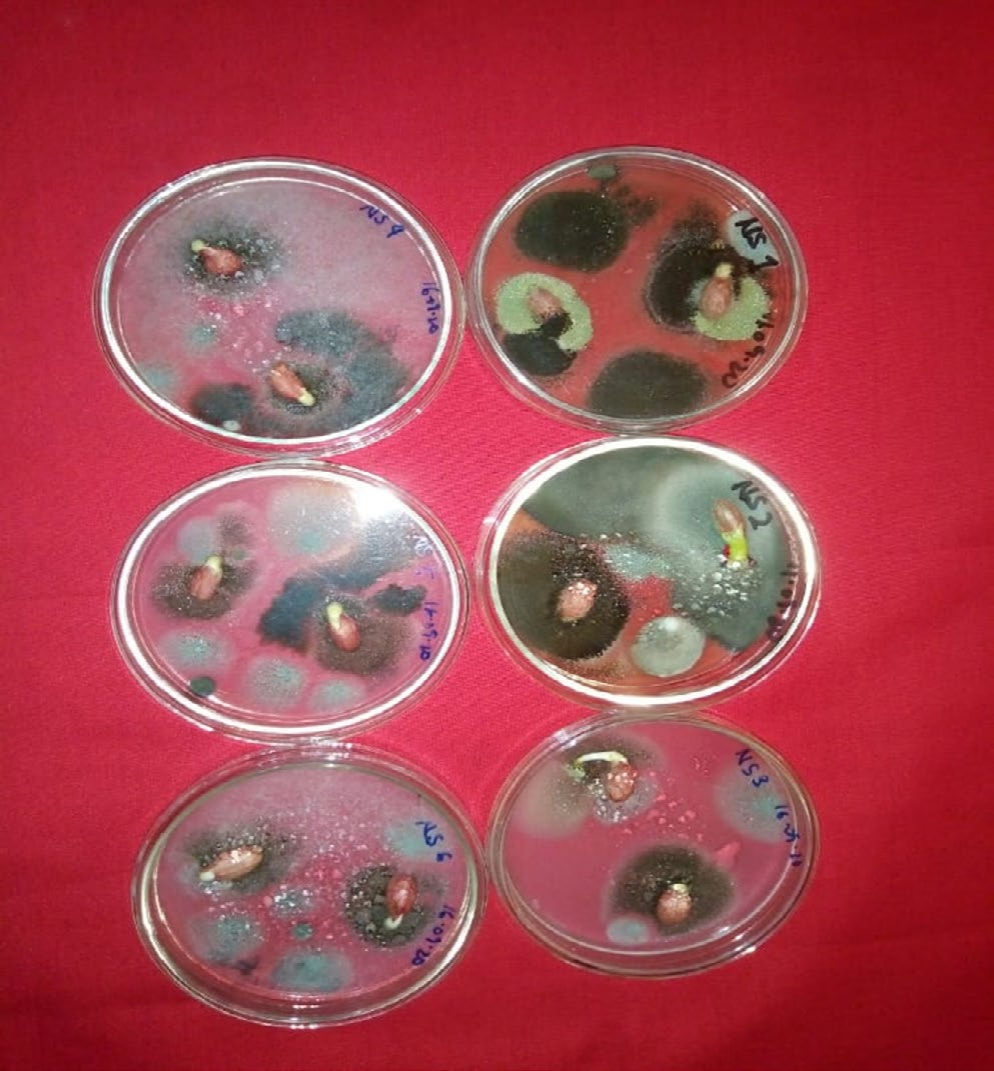
Fungi appearing on non‐surface sterilized groundnut seeds (Kumawu red variety)

**PLATE 2:**
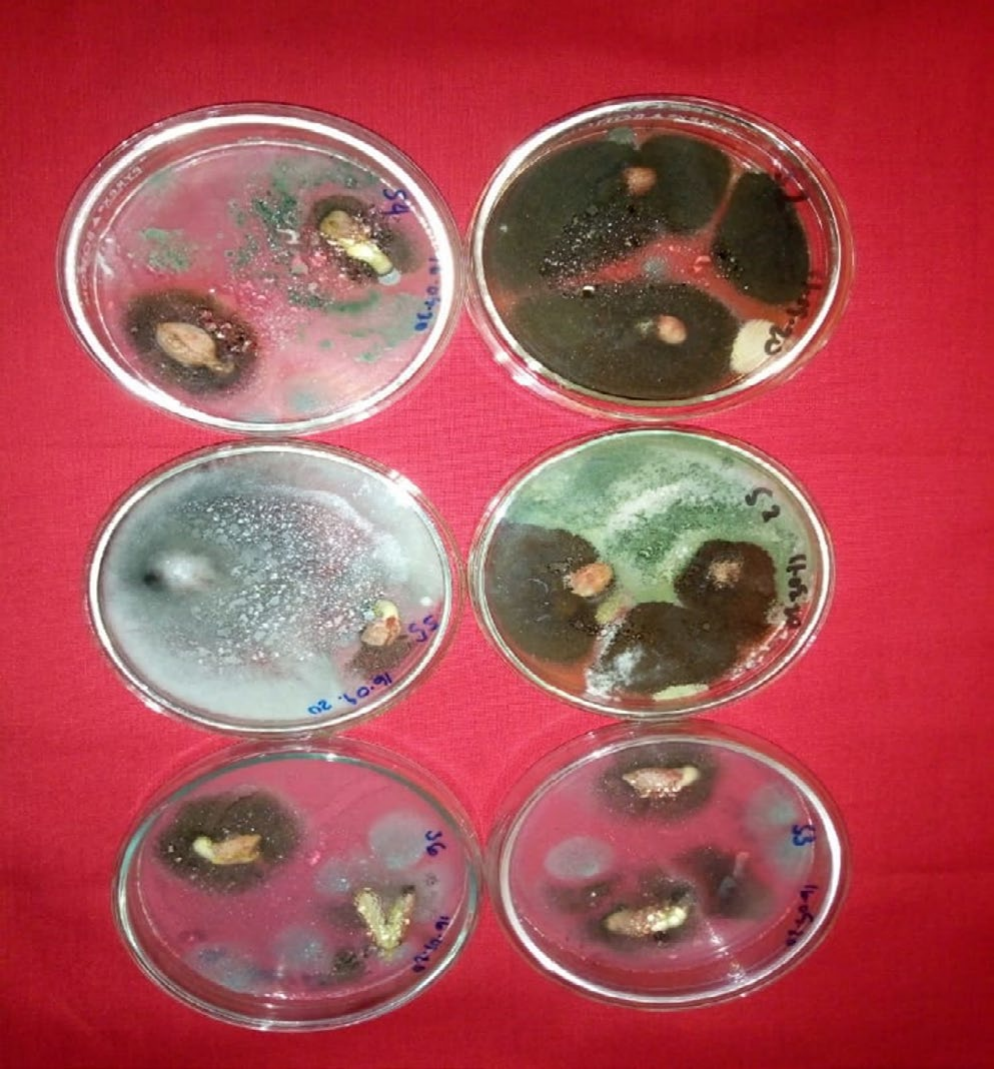
Fungi appearing on surface‐sterilized groundnut seeds (Kumawu red variety)

Clearly, the fungal species isolated in this study with toxigenic potential and human health and plant pathogenic importance were *A. niger, A. flavus, A. fumigatus, Fusarium verticillioides and Penicillium verucosum, Curvularia lunata, and Fusarium oxysporum* (Figure 3). Some of the mycobiota appearing on the non‐surface sterilized seed (Plate 1) and on the surface‐sterilized seeds (Plate 2).

## 4 DISCUSSION

Significant constraints such as drought, fungal (rusts and leaf spots), bacterial, viral diseases, as well as insect pests (leaf miners and aphids) affect groundnut production (Okello et al., 2010, 2013). Proper storage of groundnut after harvest is one maintained under clean, dry conditions with low kernel moisture content of about 8% and a low temperature with protection from insects and infestation to avoid contamination and aflatoxin formation (Torres et al., 2014). Generally, there is a safe moisture for all grains, pulses, and legumes which can prolong their shelf‐life and preclude fungal invasion. Moisture content 10% or higher after harvest of groundnut predisposes the nuts to aflatoxins contamination. Therefore, timely drying and maintenance of safe moisture level would achieve effective control of post‐harvest mycotoxin contamination (Torres et al., 2014). Indeed, according to FAO (2010), mold growth is generally curtailed when moisture is low and fungal spores are unable to have free water to commence de novo growth in storage of groundnut.

In these present investigations, fungal populations recorded on the media PDA, SDA, and OGYE increased only marginally from 0.9 to 2.6 log_10_ CFU/g in 6 months but was attended by a decline in fungal colonies from 12 to 5, except for a spike to 8 colonies/plate after 4 months (Figures 1 and 2). Correspondingly, moisture content of the groundnut seeds increased to 6.5% in 6 months (Figure 2, Table 1). However, this moisture value falls within the recommended safe moisture content for groundnut as noted by Torres et al. (2014). Awuah and Ellis (2002) also observed that groundnuts in Ghana dried to a moisture content of 6.6% were devoid of fungal infection visually for 6 months irrespective of the storage method, while at 12% MC infection by *Aspergillus parasiticus* was visible in jute bags unless a powder of *Syzygium aromaticum* was used as a protectant biofungicide to inhibit infection by *A. parasiticus*. It is recommended that for post‐harvest management of mycotoxin contamination (especially aflatoxin), maximum moisture content of 9% for unshelled groundnut and 7% for shelled groundnuts in Ghana should be maintained during storage at 10% ERH and 25–27°C (Awuah & Ellis, 2002).

Fungi invade and produce mycotoxins in foods and seeds prior to harvest, during and after harvest (often referred to as field fungi), and also during storage (storage fungi). In this case of groundnut, the inoculation and colonization by fungi and moisture content of the soil could be favorable for spores in the soil bank to infect seeds accentuated by favorable temperatures, relative humidity, and presumably insect infestation (Bediako, Ofori, et al., 2019; Gnonlonfin et al., 2013; Guchi, 2015). It was therefore not surprising that despite the low moisture content of the groundnut seeds under storage, 18 different fungal species belonging to 10 genera were isolated on the two mycological media (Table 2).

*Aspergillus species (A. niger, A. flavus*, and *A. terreus)* were most frequently encountered followed by *Fusarium (F. oxysporum, F. solani*, and *F. verticillioides), Trichoderma (T. harzianum* and *T. viride)*, and *Rhizopus (R. oligosporus* and *R. stolonifer)* (Table 2). Even though only a single species of *Penicillium (P. verrucossum*) was isolated, it is of pathological importance. Surface sterilization of seeds before plating was to remove external contamination. However, some species which appeared in the Petri plates with unsterilized seeds were same as on the surface‐sterilized seeds (Table 2, Plates 1 and 2), indicating that they were seed‐borne from the field. These were *A. niger, A. flavus, A. terreus, F. solani, T. viride, C. herbarum*, and *F. verticillioides*.

Antibiosis is a biological interaction between two or more organisms (especially microorganisms which is detrimental to at least one of them; it can also be an antagonistic association between an organism and the metabolite substance produced by another). This phenomenon of antibiosis is common among fungal species in a closed ecological niche and be responsible for the phenology of the fungal species resident in the groundnut seeds stored for 6 months. There were about five succession patterns of infection shown by the contaminating fungi (Figure 3) and this data corroborated by the preponderance of species and the disappearance of some shown in Table 3. Markwei (1976) found three patterns of infection as compared to five in this present study. Markwei (1976) also used two groundnut varieties, Florispan runner and Kumawu red from Volta Region (also used in this present study). According to Markwei (1976), the preponderance of *A. flavus* and *A. niger* on the samples increased with time. This is at variance with our findings with five patterns of infection where *A. flavus* and *A. niger,* although preponderant, decreased throughout the sampling period (Table 3).

Awuah and Kpodo (1996) sampled unspecific varieties of groundnuts from 21 markets in 10 Regions of Ghana. They found *A. flavus* (12.8%–31.7%), *A. parasiticus* (0%–0.24%), *A. niger* (34.0%), *A. candidus* (1.4%), *A. tamarii* (3.93%), *A. alutaceus* (=*A. ochraceus*, 5.26%), *Fusarium spp*. (1.45%), *Penicillium* (5.19%), *Mucor* (2.3%), *Trichoderma spp*. (0.2%), *Rhizopus stolonifer* (12.0%), and unidentified fungi (11.72%), some of which were encountered in our present study. In a recent study, Bediako, Dzidzienyo, et al. (2019); Bediako, Ofori, et al. (2019) reported the incidence of fungal flora in unspecified groundnut varieties growing in the Ashanti, Upper East, Upper West, and Northern Regions of Ghana. *A. niger* (39.9%) and *A. flavus* (26.3%) constituted the most predominant species isolated. This agrees with our findings. In this study, however, we report for the first time the incidence of *A. fumigatus* on groundnuts in Ghana. Bediako, Dzidzienyo, et al. (2019); Bediako, Ofori, et al. (2019) also recorded *Collectotrichum* (13.3%), *Rhizopus* (14.8%), *Penicillium* (5.4%), *Curvularia* (0.2%), and *A. alutaceus* (=*A. ochraceus*), some of which were also isolated. Clearly, the soil type, environmental conditions, as well as the variety of groundnut grown may influence the structure and phenology of the community (Li et al., 2018; Waliyar et al., 2007; Wang et al., 2017).

The preponderant fungal species isolated in this study with toxigenic potential, human health, and pathological importance were *A. niger, A. flavus, A. fumigatus, Fusarium verticillioides, F. oxysporum, Curvularia lunata*, and *Penicillium verrucosum*. Basically, three major genera of fungi were identified to produce mycotoxins here. This includes *Aspergillus, Fusarium,* and *Penicillium,* although other genera also produce toxic compounds (Mohammed et al., 2013). *Aspergilli* and *Penicilli* are linked to agricultural commodities during post‐harvest storage (Agriopoulou et al., 2020; Balendres et al., 2019).

Groundnut is an annual leguminous crop and plays an important role in food and nutrition security as a valuable source of protein, fats, energy, and minerals. It is also an income‐generating commodity for sub‐Saharan Africa and Asia (Diop et al., 2004). It is the 13th most important food crop worldwide (Reddy et al., 2011). However, the nutritive value of groundnut is eroded by mycotoxin contamination. Oils from groundnuts are often downgraded of its seeds are contaminated with fungi and subsequently mycotoxins of health and pathological importance (Jolly et al., 2009).

Varietal differences in *A. flavus* infection and aflatoxin production in foods have been documented (Okello et al., 2010, 2013; Upadhyaya, 2005; Upadhyaya et al., 2002; Waliyar et al., 2016). This probably partly explains the differences in susceptibility of the Ghanaian groundnut varieties to infection by *A. flavus,* especially those obtained from the Volta and Oti Regions of Ghana. Future studies will focus on varietal differences in susceptibility to potential mycotoxigenic fungi from the different agro‐ecological zones of Ghana.

Although *A. niger* is not known to produce aflatoxins, it possesses the ability to produce other toxins such as ochratoxin A, malformin, and nigerone (Siddiquee, 2018; Wagacha et al., 2013; Wang et al., 2016). Ochratoxin A is also produced by *A. alutaceus* (=*A. ochraceus*) and *A. carbonarius* (Siddiquee, 2018). Ochratoxin A is lipid soluble and is not excreted efficiently and thus accumulate in meat which exposes humans to health risk after consuming contaminated meat (Denli & Perez, 2010).

*Penicillium verrucosum* was among the frequently isolated contaminants in stored groundnuts in this present study. *Penicillium* is in the Deuteromycetes made up of diverse fungal genera of the ascomycetous fungi and contains more than 350 species. More than 80 *Penicillium* species are documented toxin producers (Agriopoulou et al., 2020). Majority of these are of significance in food safety while the rest are not. Most important are ochratoxin A, citreoviridin, penitrem A, roquefortine, and secalonic acids. The genus has major importance in the natural environment as well as food and drug production industry. Citrinin is a mycotoxin initially isolated from *P. citrinum*. Nonetheless other species such as *P. miczynski, P. hirsutum, P. verrucossum* (also isolated in this study), *P. westling, P. expansum, P. stechii*, and *P. cyclopium* have been reported to produce citrinin (Kumar et al., 2018). *P. verrucosum* and *P. viridicatum* produce ochratoxins, cyclopiazonic acid, penicillic acid, and citrinin. In higher latitudes, for example, Canada, *P. verrucosum* is the primary producer of ochratoxin A (Amézqueta et al., 2009) and this toxin is comparatively 10 times more toxic than citrinin (Amézqueta et al., 2009).

*Fusarium* species isolated from groundnut seeds produce several mycotoxins such as biologically active trichothecenes, which when ingested in high concentrations cause vomiting and diarrhea in humans. Trichothecenes are also associated with reduced weight gain and immune dysfunction in animals (Huang et al., 2019). Zearalenone causes human uterotrophic (anti‐reproduction) effects in animals and pigs (Agriopoulou et al., 2020). Another *Fusarium* species, *F. verticillioides* (*F. moniliforme*), isolated from groundnuts in the study produces fumonisin, which have neurotoxic effect in animals and is associated with esophageal cancer in sub‐Saharan Africa (Bennett & Klich, 2003; Chain et al., 2018, 2020).

*Aspergillus fumigatus* was one of the predominant fungal contaminants in the Kumawu red groundnuts from the Volta Region of Ghana isolated throughout the 6‐month sampling period (Figure 3, Table 2). *A. fumigatus* produces a mycotoxin called fumagillin (Guruceaga et al., 2018, 2020; Raffa & Keller, 2019). Fumagillin has activity on its target, the methionine peptidase type 2 (met AP2 enzyme). The same fungus also produces several mycotoxins such as gliotoxin and pseudoritin (Fallon et al., 2010; Guruceaga et al., 2020). Fumagillin is able to inhibit the function of neutrophils in blood inducing cell death in erythrocytes and also plays a role in the damage of epithelial cells which open the way for fungal invasion (Gayathri et al., 2020; Guruceaga et al., 2020). Therefore, the presence of *A. fumigatus* in groundnut in Ghana cannot be taken lightly and discounted as it has serious health implications and toxic effects on human function such as metabolism (Guruceaga et al., 2018, 2020; Raffa & Keller, 2019). These findings open a new direction of study to ascertain the presence of fumagillin in samples of stored groundnuts infected with *A. fumigatus* in Ghana. Furthermore, there are records in pertinent literature that *A. fumigatus* also produces other mycotoxins such as fumitremorgans, verruculogen, and gliotoxin and also caused aspergillosis in both human and animals (Rhodes, 2006), pulmonary aspergillosis (lung), aspergilloma (fungal ball), skin and nail infection, as well as eye and ear infections (Pitt & Hocking, 2009).

There was another interesting observation of pathological importance. *Fusarium oxysporum*, which was initially visibly absent on the surface, was detected after 1 month and persisted in the seeds for 6 months (Figure 3). *F. oxysporum* is a well‐known plant pathogen causing severe damage in many agricultural crops, both in the field and during post‐harvest storage (Mondani et al., 2021; de Lamo and Takken, 2020). Interaction between plant and root‐colonizing *F. oxysporum* can be natural, beneficial, or detrimental to the host. *F. oxysporum* is famous for its ability to cause wilt, root, and fruit rot in many plant species including many agriculturally important crops (Dean et al., 2012). This fungus ranks among the 10 most devastating fungal plant pathogens worldwide (Dean et al., 2012) and wilts are a major threat for agricultural productivity (Fisher et al., 2012). The association of *Fusarium oxysporum* with stored groundnuts should give a cause for alarm and attract important attention especially if the seeds are meant to be used as seed bank for the next season's food cultivation. Future studies will examine the pathology of this fungus in the groundnut ecosystems with the view to assessing the pathogenicity of *F. oxysporum* in the groundnut varieties cultivated in Ghana.

*Curvularia lunata* (Ascomycota; order Pleosporales) found in soil was also isolated in this study (Tables 2 and 3; Figure 3). *C. lunata* has been identified causing brown leaf spots in Pakistan (Majeed et al., 2016); blight disease of rice in India (Kamaluddeen & Abhilasha, 2013); leaf spot of *Brassica rapa* ssp. *pekinensis* in Thailand (Wonglom et al., 2019); *Curvularia* leaf spot of corn (*Zea mays*) in USA (Anderson et al., 2019; Garcia‐Aroca et al., 2018); and a plant pathogen in China (Chang et al., 2020; Liu et al., 2014). This fungus has been reported as pathogenic to crops of economic importance in Africa including Burkina Faso, Egypt, Ethiopia, Democratic Republic of Congo, Gambia, Ghana, Guinea, Kenya, Nigeria, Senegal, Sierra Leone, South Africa, Sudan, and Tanzania (Lorrain et al., 2019). In Ghana, *C. lunata* has been reported to infect some cereal grains; sorghum (Nutsugah et al., 2007) and maize (Hackman, 1995).

The genus *Curvularia* is also a dematiaceous fungus in the environment worldwide, mainly in tropical regions (Revankar, 2007). The genus *Curvularia* comprises of several species, three of which are ubiquitous and cause several types of infection in both immunocompetent and immune‐compromised horses, namely *C. lunata, C. pallescens*, and *C. geniculata*. *C. lunata* is the most commonly reported in human infections (Brandt & Warnock, 2003; More et al., 2019). *C. lunata* causes allergenic nasal polyposis and occasionally eosinophilia. Building up population of *C. lunata* in storage bags may predispose workers to inhalation of spores during handling in storage rooms.

## 5 CONCLUSIONS AND RECOMMENDATIONS

Data from this present study have shown that Kumawu red groundnut seeds from the Volta and Oti Regions harbored 18 fungal species belonging to 10 genera. *Aspergillus* species (*A. niger, A. flavus, A. fumigatus, A. ustus*, and *A. terreus*) predominated over all the others encountered followed by *Fusarium* (*F. oxysporum, F. verticillioides, F. solani*), *Trichoderma* (*T. harzianum* and *T. viride*), *Rhizopus* (*R. oligosporus, R. stolonifera*), *Cladosporium herbarum, Curvularia lunata, Penicillium verrucosum, Paecilomyces variotii, Rhodotorula mucilaginosa,* and *Sporendonema casei*. Some of the species were seed borne (*A. niger, A. flavus, A. terreus, A. fumigatus, F. solani, F. verticillioides, T. viride, C. herbarum*, and *Curvularia lunata*) and were isolated from both surface sterilized and non‐surface sterilized groundnut seeds.

Mycotoxin analysis of groundnuts was concentrated on aflatoxin group and to a larger extent on other equally potent mycotoxin. Our results show that a wider range of mycotoxigenic fungi contaminate groundnuts grown in the study area. This suggests the need to widen the scope of mycotoxin analysis to cover possible mycotoxins such as fumagillin (*A. fumigatus*), ochratoxin A (*P. verrucosum*), and fumonisin (*Fusarium spp*.), which have demonstrated to have human health implications. Furthermore, *F. oxysporum* isolated in this study ranks among the 10 most destructive wilt fungal pathogens worldwide (Dean et al., 2012; Fisher et al., 2012). Its presence in groundnuts seed could decimate crop productivity in the field.

*Curvularia lunata* found in soil bank has been recorded as pathogenic to many crops of economic importance in Africa including Ghana (Kusai et al., 2016) and also is a human pathogen as well (Kiss et al., 2020). Future studies will address this aspect of the shelf‐life of the stored legumes like groundnut. Finally, there are varietal differences in susceptibility of groundnuts to fungal infection and mycotoxin formation in the field and in storage. This study only scratches at the surface of a wider crop productivity and seed storage pathology problem and it opens a relevant gate for future research on safety of stored groundnut, especially so far as toxin contamination and seed viability are concerned.

## CONFLICT OF INTEREST

The authors declare that they do not have any conflict of interest.

## AUTHOR CONTRIBUTIONS

**Nii Korley Kortei:** conceptualization (lead) ; methodology (supporting); writingOriginalDraft (equal); writingReviewEditing (equal). **Rachel Adinorkie Tetteh:** dataCuration (equal) ; investigation (equal); projectAdministration (equal). **Michael Wiafe‐Kwagyan:** conceptualization (equal) ; methodology (equal); writingOriginalDraft (equal); writingReviewEditing (equal). **Denick Nii Kotey Amon:** dataCuration (equal); methodology (equal); resources (equal). **George Tawia Odamtten:** conceptualization (equal); methodology (equal); supervision (lead); writingReviewEditing (equal).

## Data Availability

The data that support the findings of this study are openly available in this journal.
